# Application of a Modified Healthy Eating Index (HEI-Flex) to Compare the Diet Quality of Flexitarians, Vegans and Omnivores in Germany

**DOI:** 10.3390/nu14153038

**Published:** 2022-07-24

**Authors:** Anja Bruns, Mattea Mueller, Inga Schneider, Andreas Hahn

**Affiliations:** Institute of Food Science and Nutrition, Leibniz University of Hannover, 30159 Hannover, Germany; mueller@nutrition.uni-hannover.de (M.M.); schneider@nutrition.uni-hannover.de (I.S.); hahn@nutrition.uni-hannover.de (A.H.)

**Keywords:** flexitarians, HEI-flex, Healthy Eating Index, diet quality, vegans, plant-based alternatives

## Abstract

Interest in plant-based nutrition has steadily increased in the western world in the recent years. The number of people following a meat-reduced, flexitarian diet is growing continuously. However, little is known about the diet quality of flexitarians compared to vegans or omnivores. Therefore, in this cross-sectional study, the food intake of 94 participants aged between 25–45 years was recorded via a validated food frequency questionnaire and 28 self-designed questions about the consumption of plant-based alternatives. An adapted Healthy Eating Index, HEI-flex, was developed to evaluate the diet quality of flexitarians, vegans and omnivores. Higher score points (SP) of the HEI-flex are associated with higher compliance with the official diet recommendations (V_max_ = 100 SP). Finally, flexitarians scored significantly more highly when compared to omnivores (54 ± 8 vs. 47 ± 9 SP; *p* = 0.008) but lower than vegans (54 ± 8 vs. 61 ± 10 SP; *p* = 0.010). The results showed that the HEI-flex is a useful tool for assessing and comparing the diet quality of flexitarians, vegans and omnivores. Despite the consumption of highly processed plant-based alternatives, reduction in meat and meat products seems to be accompanied by increased overall diet quality.

## 1. Introduction

Plant-based diets are becoming increasingly popular in the Western world [1,2,3]. Although vegans (V) do not eat any food of animal origin, the food choices of vegetarians are very heterogeneous. Certain animal products are included or excluded depending on the form of vegetarianism. Additionally, there is a rising prevalence of the flexitarian (FX) diet in Western countries, which is broadly characterized by a primarily vegetarian diet pattern with occasional meat or fish consumption [4,5,6,7]. However, a generally accepted definition of flexitarianism does not currently exist. Instead, different definitions and consumption quantities of meat and meat products are discussed in various studies [8,9,10]. In Germany, the recommendations of the German Nutrition Society (DGE) define an FX diet as consuming meat or fish fewer than 4 days per week [11,12].

Interestingly, despite the rising popularity of a meat-reduced FX diet, annual meat consumption in Germany is currently only slightly declining, while the consumption of plant-based meat alternatives is growing constantly. The sales of plant-based meat alternatives overall increased by over 30% in 2021 compared to the previous year [13,14,15,16]. A consumer survey (*n* = 1000) in 2020 reported that half of the participants had purchased vegetarian or V meat alternatives in the past. Furthermore, FX households were four times more likely to consume vegetarian or V meat alternatives than omnivorous households. Plant-based alternatives, such as vegetarian/V burger patties, are suggested to be an equivalent (or better) nutritional substitute to the animal-based, conventional products in advertising [5,13,17,18,19,20,21,22,23,24].

Diet quality as a global indicator of food diversity is commonly used to estimate the degree of compliance with the dietary recommendations given. The Healthy Eating Index (HEI) is a frequently used diet quality score. It was developed in 1995 to assess the overall diet quality of the American population by integrating nutrient requirements and dietary guidelines for Americans in one single measure [25,26]. The HEI has been revised and later adapted by other countries considering country-specific guidelines [27,28,29,30,31,32,33]. The HEI has also been used in Germany in modified form, based on official dietary recommendations, for example, as HEI-Epic (relationship between dietary intake and cancer, *n* = 5465) or HEI-NVSII (dietary intake of organic buyers and non-organic buyers, *n* = 13,154) [34,35,36]. However, neither the great variety nor the nutritional quality of plant-based alternatives has been integrated into a diet quality evaluation based on a HEI yet [35,36,37,38].

When comparing the quality of a meat-reduced FX to a V or omnivore (OMN) diet, different dietary recommendations must be considered in order to avoid point losses for a reduced intake.

Therefore, the aim of this study was to develop an adapted HEI, termed HEI-flexible (HEI-flex), which enables the evaluation of the diet quality of FX, V and OMN. Therefore, the official food frequency questionnaire (FFQ) was extended with questions regarding plant-based alternatives, and the new HEI-flex was conceptualized using different sources for official dietary guidelines in Germany (e.g., V diet pyramid). Finally, a cross-sectional pilot study was performed using the HEI-flex score based on food intake data derived from FX, V and OMN.

## 2. Materials and Methods

### 2.1. Study Design, Participants and Procedure

A cross-sectional pilot study including 94 participants was conducted from January to June 2020 using recruitment by flyers, social media and online communities.

The eligibility of participants was assessed using an online questionnaire to define their dietary patterns. This self-defined diet was confirmed during a subsequent telephone interview specifically focused on the daily intake of meat and meat-derivates to accurately differentiate between FX and OMN.

The present study included either participants who (a) maintained a flexitarian (FX) diet (i.e., plant-based diet with an occasional consumption of meat and meat products of ≤50 g/d) or (b) maintained a vegan (V) diet (i.e., avoidance of any food of animal origin) or (c) maintained an omnivore (OMN) diet (i.e., mixed diet with regular consumption of meat and meat products of ≥170 g/d).

The consumption limits for meat and meat products for FX were derived from the lower consumption recommendations of the DGE (300–600 g/week) [12]. The minimum intake of meat and meat products for OMN was based on the average consumption in Germany in 2019 (62 kg/year/person) [20,39,40].

Furthermore, all participants had to adhere to their respective diet for at least 1 year and had to be healthy, nonsmokers, aged between 25 and 45 years and with a body mass index (BMI) between 20 and 28 kg/m^2^.

Interested subjects with consumption rates of meat and meat products from ≥50 g/d ≤ 170 g/d were not included to achieve a clear differentiation between the three groups.

Other exclusion criteria were acute infections, several chronic diseases, regular use of laxatives, drugs, alcohol or medication abuse.

If eligible, the participants were invited for a study visit. On the latter, anthropometrics was measured, fasted blood samples were taken and several questionnaires were filled out.

This study was conducted at the Institute of Food Science and Human Nutrition at Leibniz University Hanover, Germany, according to the guidelines of the Declaration of Helsinki. Ethical approval was provided by the Ethics Committee of the Medical Association of Lower Saxony (Hanover, Germany). The study was registered in the German Clinical Trial Register (DRKS 00019887). All subjects provided written informed consent. Participants were matched by age and gender within their respective group and across all three groups.

### 2.2. Anthropometrics, Blood Samples and Measurement of Blood Pressure

The height and weight of the participants were measured on the examination day. The waist and hip circumference were determined using a tape measure. BMI was calculated according to the standard formula [41,42]. The body composition and the basal metabolic rate (kcal/d) were assessed using multifrequency bioelectrical impedance analysis, according to the manufacturer’s recommendations, by trained nutritionists of Nutriguard M (Data Input Company, Darmstadt, Germany).

After overnight fasting (≥12 h), a licensed doctor took blood samples, obtained by puncture of an arm vein from each participant. On the same day, all samples were deep frozen transferred (−18 °C) and determined in the accredited and certified laboratory of Clinical Chemistry, Hannover Medical School, Germany. Blood pressure was measured, according to the manufacturer’s recommendations, by trained nutritionists using Visomat (UEBE, Medical Wertheim, Külsheim, Germany).

### 2.3. Physical Activity

Physical activity was assessed using a validated German Freiburger Questionnaire [43]. This questionnaire considers all health-relevant activities, from which the daily energy expenditure in kcal/day was estimated. Energy expenditure and the basal metabolic rate were added to determine total energy expenditure per day and participant.

### 2.4. Dietary Habits

Food intake rates were assessed using the validated FFQ of the RKI, Germany [44]. However, the large variety of plant-based alternative products has not yet been ascertained. Consequently, 28 additional questions covering plant-based alternative products—low or high processed—were added (Appendix A).

Furthermore, prior to the study visit, participants were asked to fulfill a three-day diary record over three consecutive days, including two weekdays and one weekend day. These records were checked by nutritionists for completeness, readability and plausibility. Ambiguities were clarified with the participants if necessary. Data from these dietary logs were processed using PRODI^®^ 6.12, Nutri-Science GmbH, Freiburg, Germany (Organizational software for nutrition counselling based on the German Federal Food Code 3.02) to derive the daily intake of macro- and micronutrients of each participant.

### 2.5. Development of HEI-Flex

The HEI-flex is based on the validated HEI-2015 [45,46,47,48] and was adapted via the following major points.

#### 2.5.1. Assignment of Foods into HEI-Flex Components

In principle, the HEI-flex is based on the food intake data derived from the validated German FFQ and 28 additional questions regarding plant-based alternatives (Appendix A). These food quantities were standardized to grams (g), milliliters (ml or centiliters (cl) per four weeks (28 days), according to the specifications of the RKI [44,49]. In the next step, the mean daily intake was calculated using the following formula:


$$\mathrm{Mean}\;\mathrm{daily}\;\mathrm{intake}\;=\;\frac{\mathrm{consumption}\;\mathrm{frequency}\;\times \;\mathrm{portion}\;\mathrm{amount}\;\mathrm{in}\;\mathrm{g/ml/cl}}{28\;\mathrm{days}}$$

These mean daily intake rates of all foods queried were allocated to 14 components, which were based on the DGE guidelines, the macronutrient content and the degree of processing [12,14]: (1) beverages, (2) vegetables, (3) fruit, (4) protein sources, (5) carbohydrate sources, (6) whole meal, (7) nuts and seeds, (8) processed meat and plant-based meat alternatives, (9) milk and dairy products and plant-based dairy alternatives, (10) alcohol, (11) high-energy density foods (sweet), (12) high-energy density foods (fat), (13) drinks with high-energy density, and (14) fats and oils and plant-based fat substitutes (Table 1).

#### 2.5.2. Integration of Various Official Dietary Recommendations

Ratios between intake and intake recommendation had to be formed to calculate the HEI-flex score values for each component. Therefore, the German nutritional guidelines of DGE were used for FX and OMN, and, in addition, recommendations of the Giessen Vegan Pyramid for V [12,50]. If no German recommendations were available (components 10 and 13), guidelines from WHO as well as the dietary guidelines for Americans, respectively, were considered (Table 1) [26,51].

As Table 1 shows, the ratio of the mean daily intake and the official intake recommendation [12,50,51] was calculated using the following formula for all components, except components (11) high-energy density foods (sweet) and (12) high-energy density foods (fat):


$$(\frac{V}{E})X\;=\;\frac{\mathrm{Mean}\;\mathrm{daily}\;\mathrm{intake}\;\mathrm{of}\;\mathrm{the}\;\mathrm{component}}{\mathrm{dietary}\;\mathrm{guideline}\;\mathrm{recommendation}}$$

*V* = Amount consumed in g.

*E* = Official recommendation in g.

#### 2.5.3. Using Individual Energy Expenditure Values

Regarding the calculations of foods listed in components 11 and 12 (foods with high-energy density), limits of total energy expenditure in % were used in accordance with comparable HEI-2015 components (“Added Sugars” and “Saturated Fats”) [47,52]. Therefore, the energy content of the foods consumed within these components was determined and was put in relation to the individual total energy expenditure of each participant (Section 2.5.4 and Table 1).

#### 2.5.4. Calculating Principles and Scoring

In order to be able to make an evaluation of the ratio of the mean daily intake and the official intake recommendation, four calculation principles were applied for the scoring of all components: the adequacy principles I and II and the moderation principles III and IV (Appendix B). Which principle was used depended on whether the food group components had a recommended or tolerated intake, according to the dietary recommendation.

The adequacy principle I was applied for foods listed in components 1–3; its recommendations focus on the minimal amount that should be consumed daily. Point losses occurred proportionally if the consumption rate was too low up to the minimum required intake amount.

The adequacy principle II was used to calculate the components where a consumption range was given (components 4–9 and 12). Point losses occurred proportionally if the supply quantity was too low, and if it was higher than the maximum quantity.

The moderation principle III was applied for components 13 and 14, as it is recommended to consume as little as possible. No consumption of foods from these components resulted in the maximum value (V_max_ = 100 SP). The higher the consumption rates, the higher the point losses proportionally.

The moderation principle IV was used for components 10 and 11 as a tolerated consumption range is used. Point losses occurred proportionally when the tolerated maximum quantity was exceeded.

All components within the HEI-flex were weighted equally because proportional point losses were recorded by under- and overconsumption via the calculating principles mentioned above.

If a component contains different subgroups, the consumption rate of each subgroup was individually compared with the respective recommendations and SP were calculated. The average SP value of all subgroups forms the SP for the parent component (cf. Table 1).

Finally, a maximum of 100 points could be earned per component. Hence, a maximum of 1400 points were achievable for 14 components, which was divided by 14 to determine the total score points (SP). Thus, the HEI-flex scales from 0 to 100 SP, whereby a higher value reflects a higher adherence of official dietary recommendations.

### 2.6. Statistical Evaluation

Results are presented as median (25th, 75th percentile) or mean ± standard deviation (SD). The normal distribution of data was initially checked visually and subsequently with the Kolmogorov–Smirnov test. The one-way analysis of variance (ANOVA) was used with normal distribution to assess differences between the three diets. If the data set was not normally distributed, the Kruskal–Wallis test was used. The *post hoc* test with Bonferroni correction was performed if there were significant differences. The chi-square test was used to compare frequencies between the participants. The Mann–Whitney U test was used for calculations conducted between men and women.

Correlations between HEI-flex data and values from the food diaries were calculated using Spearman’s Rho test. Moreover, *p*-values ≤ 0.05 were interpreted as statistically significant in this study.

Microsoft Excel 2019 MSO, version 1808, was used for the HEI-flex calculations. Statistical analyses were performed using SPSS software IBM SPSS Inc Statistics 28.0.1.0 (Chicago, IL, USA).

## 3. Results

### 3.1. Characterization of the Study Population

A total of 32 FX, 33 V and 29 OMN were included in the study (Table 2). The participants did not differ significantly regarding age or sex distribution. The duration of adhering to the respective diet differed greatly, however, the minimum duration was at least 1 year as defined by the inclusion criteria. Significant differences also existed in the BMI among all study participants across the three diets (*p* = 0.005). As Table 2 shows, FX had a lower BMI in kg/m^2^ when compared to OMN (FX 22 (21–25) vs. OMN 25 (23–27); *p* = 0.003), yet the BMI of V vs. OMN or V vs. FX was not significantly different. Comparing the energy expenditure of health-related activities (kcal/week), FX had neither higher values than OMN nor less than V, and it was not significant, respectively. However, the calorie requirement of V was more than twice as high as OMN (V 5793 (3111–8168) vs. OMN 2681 (1385–4286); *p* = 0.002).

### 3.2. Diet Quality Score

**Figure 1:**
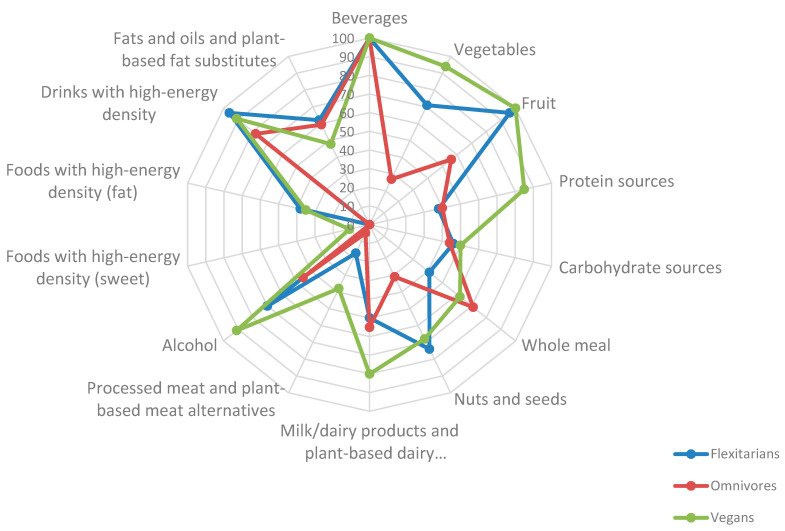
Total score points of flexitarians, vegans and omnivores subdivided by HEI-flex components in a network graph. Plot of the median score values of flexitarians, vegans and omnivores subdivided by the 14 components of the HEI-flex (SP_min_ 0/SP_max_ 100) in a network graph. HEI-flex = Healthy Eating Index—flexible.

As shown in Table 3 and Figure 1, FX scored significantly higher than OMN (FX 54 ± 8 vs. OMN 47 ± 9 SP, *p* = 0.008) but significantly lower than V (FX 54 ± 9 vs. V 61 ± 10 SP, *p* = 0.010). Regarding differences between sexes (Table 3), the total SP of men and women do not differ significantly (SP_female_ 56 (±11) vs. SP_male_ 52 (47–58); *p* = 0.124).

Significant differences were found in the component “Vegetables” between FX and OMN (FX 71 SP (3699) vs. OMN 27 SP (17–38); *p* = 0.011) and between V and OMN (V 94 SP (48–100) vs. OMN 27 SP (17–38); *p* ≤ 0.001). Particularly low SP were found in OMN men (OMN_men_ 26 SP (14–38).

Calculations with the “Fruit” component resulted high SP for FX and V (FX 96 SP (62–100) vs. V 100 SP (65–100); *p* = 1.000). The OMN had lower SP than FX (OMN 56 SP (36–100) vs. FX 96 SP (62–100); *p* = 0.141) and significantly lower SP than V (OMN 56 SP (36–100) vs. V 100 SP (65–100); *p* = 0.020).

Moreover, FX and OMN achieved low SP in “Protein sources” (FX 38 SP (29–50) and OMN 40 SP (34–53); *p* = 1.000). By contrast, V reached significantly higher SP than FX (V 85 SP (76–98) vs. FX 38 SP (29–50); *p* ≤ 0.001) and OMN (V 85 SP (76–98) vs. OMN 40 SP (34–53); *p* ≤ 0.001). Within this component, FX_female_ achieved the lowest values of all three groups (FX_female_ 32 SP (22–47)).

Considering “Milk/dairy products and plant-based dairy alternatives”, FX scored significantly lower compared to V (FX 50 SP (28–67) vs. V 80 SP (45–100); *p* = 0.006) and lower, but not significantly, compared to OMN (FX 50 SP (28–67) vs. OMN 55 (47–89); *p* = 0.271).

Regarding the component “Alcohol”, FX achieved SP between V and OMN (FX 70 SP (19–96) vs. V 91 SP (41–100) vs. OMN 46 SP (17–77); *p* = 0.018), whereby the SP of V were 21% higher than FX, and the SP of OMN were 24% lower than FX. Consequently, a significant difference for V compared to OMN was observed (V 91 SP (41–100) vs. OMN 46 SP (17–77); *p* = 0.013).

Furthermore, FX and OMN had similar low SP in “High-energy density foods (sweet)”. Therefore, no median SP could be given for either of these diets (FX 0 SP (0–33) vs. V 0 SP (0–10); *p* = 0.722). The V consumed these high-calorie, low-fiber products almost as frequently; consequently, only low SP could be awarded (V 11 SP (0–52)). Based on these values, significant differences were found between V and OMN (V 11 SP (0–52) vs. OMN 0 SP (0–10); *p* = 0.037).

The FX scored slightly higher than V in the component “High-energy density foods (fat)” but overall at a low level (FX 38 SP (0–100) vs. V 35 SP (0–85); *p* = 1.000). Moreover, OMN did not score any points in this component, which differed significantly from FX (OMN 0 SP (0) vs. FX 38 SP (0–100); *p* = 0.012) and V (OMN 0 SP (0) vs. V 35 SP (0–85); *p* = 0.010).

In addition, no significant differences were found in “Beverages”, “Carbohydrate sources”, “Whole meal”, “Nuts and seeds”, “Processed meat and plant-based meat alternatives”, “Drinks with high-energy density” and “Fats and oils and plant-based fat substitutes” between the three groups.

### 3.3. Indications for Relative and Construct Validity: Associations between Nutrient Intake of the 3-Day Food Record and HEI-Flex Calculations

In order to gain an initial insight into the relative and construct validity of the HEI-flex, the score components were correlated with the macronutrient intake based on the three-day food record (Table 4) and compared across the thirds of HEI-flex, moreover, cardiovascular risk parameters were also considered (Table 5).

Observing the first indications of relative validity, significant correlations were found between total score points of HEI-flex and the intake of “Total energy”, “Total fat” and “Water”, “Dietary Fiber”, “Sodium”, “Alcohol” with the Spearman’s rank correlation coefficient (rs) (*p*_rs_ < 0.05 respectively). The directions of these correlations implicated that higher HEI-flex SP are associated with a more beneficial macronutrient profile, i.e., a higher intake of dietary fiber and lower intake of total energy, fat, alcohol and sodium was correlated with higher diet quality according to the HEI-flex Furthermore, “Dietary Fiber” correlated positively with the component “Protein Sources”, which is partly due to the fact that plant, as well as animal based protein sources, were captured in this component.

No significant correlations could be observed between the macronutrients “Protein”, “Total fat” and “Carbohydrates” and SP of high-energy density foods and drinks because protein-, fat- and carbohydrate-rich foods went into different components of HEI-flex according to the processing or preparation method. For example, carbohydrate-rich foods from the food diary log went in different proportions into the components of “Whole meal”, as well as “Carbohydrate sources” or “High-energy density foods (sweet). Similar, protein-rich foods were assigned to either the component “Foods with high-energy density -fat-“ or “Protein sources”.

To sum up, many rs-values ranged between 0.2 and 0.3.

In addition, regarding first indications in construct validity, participants in the upper third of the HEI-flex had a higher intake of dietary fiber (*p* ≤ 0.001), but lower intake of energy, total fat, salt and alcohol. In the lower third, it was nearly inverse: there was a higher intake of energy, carbohydrates, total fat, salt and alcohol, but a lower intake of dietary fiber and water.

Regarding cardiovascular risk parameters, measured values showed the most positive tendencies within the upper third. For example, triglycerides, LDL-cholesterin and systolic blood pressure lowered with the higher third (n.s.), respectively. The decreasing insulin level with higher thirds were significant (*p* = 0.013).

## 4. Discussion

The aim of the present study was to develop an adapted HEI-2015, which enables the comparison of the diet quality of FX, V and OMN in a healthy German adult population. Therefore, a new HEI, termed HEI-flex, was developed integrating the wide range of plant-based alternatives, either low or high processed, and specific official V recommendations [12,50]. Thereby, the higher the compliance with the dietary guidelines, the higher the score points (SP) given (V_max_ = 100 SP). In summary, FX scored lower than V but higher than OMN (FX 54 SP ± 8 vs. V 61 SP ± 10 vs. OMN 47 SP ± 9); *p* ≤ 0.001).

These HEI-flex results confirm previous studies observing the higher diet quality in plant-based diets compared to OMN, based on various diet quality indices. However, many of these diet quality indices (e.g., diet quality index, Mediterranean diet score, plant-based index) often differ in terms of their research aim (e.g., associations between dietary intake and health outcomes) or scoring methodology (e.g., allocation of foods only according to positive and negative) [53,54,55,56,57,58,59,60]. Therefore, only studies based on the original HEI versions are included in the discussion below.

Several studies have determined the diet quality for different target groups (e.g., national consumer surveys, children or seniors), based on an HEI [27,37,38,61,62,63,64]. However, calculations of plant-based diets using an HEI are rare. Semi-vegetarians and V were considered in a Belgium study based on HEI-2010 only once [53]. Nutritional quality has already been determined in Germany several times based on an HEI [35,36,37,38], but so far, neither FX nor V have been explicitly studied regarding their diet quality. Furthermore, specific recommendations that apply to V have not yet been addressed in current HEI evaluations [35,37,38,50]. Therefore, the HEI-flex has integrated some important extensions and amendments, which are discussed below.

### 4.1. Development of HEI-Flex

Four key aspects are particularly focused on in the following concerning the HEI-flex development.

Firstly, the HEI-flex was based on FFQ data, including low or high processed plant-based alternatives. Thereby, the increasing consumption of these plant-based alternatives and their health quality were reflected. However, these foods have not been explicitly recorded yet [28,36,37,38,52,53,64] or have only been added to protein sources without considering the degree of processing [53].

Secondly, the components of HEI-flex considered all food groups from the official German intake recommendations for OMN (including FX) and V [12,50]. Hence, country-specific dietary habits and the particular nutritional recommendations of V have been taken into account. By contrast, other German HEI versions do not differentiate between various diets [36,38] or they integrated only a few specific German foods (e.g., whole meal bread) into the HEI-2015 component classification [37].

Thirdly, it is important to emphasize that all components in the present study were assigned the same weighting corresponding to the HEI-DEGS [38]. However, the HEI-flex differs from the original HEI-2015 and adapted German versions, which assign different maximum values to each component [35,52]. By comparison, every component in the HEI-flex may reach a maximum of 100 SP, whilst overconsumption of components with a consumption margin will be assessed with point losses. In this way, a much more differentiated assessment of the consumption rates becomes possible.

Lastly, while all other comparable HEI versions used only official standard values for total energy expenditure for calculating the scores of food components with high-energy density (sweet and fat) [37,38,52,53], the HEI-flex calculations in the present study consider the individual total energy expenditure of each participant assessed by physical activity questionnaires (cf. 2.3).

To sum up, the new developed HEI-FX integrates plant-based alternatives accounts for the processing of foods, considers specific recommendations for omnivores, as well as for vegans and uses the individual energy expenditure of each participant for calculating components with high energy density. This approach allows an evaluation of nutritional quality that can be applied to FX as well as to V and OMN (cf. Appendix C).

### 4.2. Components of HEI-Flex

The HEI-flex contains a special compilation of food components that focused especially on the wide range of plant-based alternatives and official V recommendations.

The components of the HEI-flex will be discussed in the following, pointing to conspicuous values: “Processed meat and plant-based meat alternatives” and “Foods with high density (sweet and fat)”.

It is noteworthy that a high consumption of “Processed meat and plant-based meat alternatives” (e.g., cold cuts or sausage, processed plant-based burger patties or meat alternatives) were observed across all three diets in the present study. About a quarter of all participants achieved no SP in this component, which probably also lowered the total SP. The comparable consumption rates of processed meat, fish or sausage in SP results were also roughly found in the HEI-EPIC study, although the allocation of foods into components differed partly, and no distinction between diets was made [35]. Nevertheless, V has also only moderately low SP in this component compared to FX and OMN based on HEI-flex, emphasizing that highly processed animal-based products are a crucial component in the diet quality evaluation. In terms of health, these products are associated with negative effects on cardiovascular and carcinogenic risk [65,66]. Whether high processed plant-based alternatives have similar detrimental effects on cardiovascular health as high processed animal-based products still needs to be confirmed.

Furthermore, very low SP were observed in the two components in the present study with “High-energy density foods”. The overconsumption in these components has partly contributed to the lower total HEI-flex SP in FX and OMN, compared to V. Whereby, V also achieved a low SP in both of these components (≤35 SP). However, point losses in these components generally differ from previous studies [35,38,53,55]. Perhaps the associated food groups were not asked about in such detail or were assigned to other components in the other surveys mentioned above.

Finally, regarding the SP of all fourteen HEI-flex components, it is noticeable that FX did not score significantly higher than V or significantly lower than OMN in any component. These results are essentially in line with the Belgium study of Clarys et al. [53]. This is interesting when considering that neither the number of components matches the allocation of the plant-based alternatives nor the component-weighting and valuation principles. Hence, HEI-flex calculations and Clarys et al. (2014) show that an animal-reduced diet is associated with higher SP values.

### 4.3. Total Scoring with HEI-Flex

The total SP of FX, V and OMN in the present study were generally similar to the results observed by Clarys et al., who also reported higher SP for V when compared to semi-vegetarians and OMN based on HEI-2010 [53].

However, the total SP of this study were generally slightly higher, compared to the present study. On the one hand, these differences could be due to the subdivision of low or high processed foods into different components in HEI-flex, and, on the other hand, soft drinks with a high-energy density and alcohol were also included in the calculations in contrast to the Belgium study. Point losses in these components were probably one reason that the total SP of HEI-flex were significantly lower than other calculations based on HEI-2010, respectively, HEI-2015 [27,33,37,38,53,61]. Moreover, the majority of participants in Clarys et al. (2014) were female—the gender ratio in the present study was balanced. The higher SP of female compared to male study participants was also found in other comparable German studies based on an HEI [35,37].

Interestingly, the SP values of OMN Japanese women and men were quite similar to OMN American women and men based on HEI-2015 [61]. However, the individual SP of the components between the two populations were considerably different, presumably due to the country-specific foods, yet total scores were nearly the same. Equally, a similar interindividual variation was observed in the present study, since the standard derivations were quite wide in several components (e.g., Nuts and seeds, FX, 74 SP (14–100)).

In summary, national dietary habits, various food components, their individual weighting, different valuation principles and resulting maximum SP make a comparison of various HEI versions difficult. Moreover, due to the aspects already mentioned, a direct SP comparison does not appear to be purposeful and should be weighed up on a case-by-case basis.

### 4.4. Strengths and Limitations of the Study

The present study showed that the HEI-flex is a useful tool to evaluate the diet quality of diverse long-term dietary patterns by combining recommendations of mixed diets (FX and OMN) and V, considering the intake of plant-based alternatives and high processed foods.

Furthermore, the HEI-flex calculations ensure that the lack of consumption of certain food groups, for example, dairy products, and meat or plant-based alternatives, did not result in any disadvantages in scoring. However, under- or overconsumption in certain components also led to point losses. Another advance of the HEI-flex compared to the HEI-2015 is the inclusion of beverages and alcohol (“Beverages”, “Drinks with high-energy density” and “Alcohol”) in three independent components, which ensured a more differentiated assessment of diet quality. In addition, the HEI-flex can also calculate SP by gender (cf. Appendix C).

A limitation of the study is its cross-sectional design, which restricts conclusions regarding causality. Moreover, most of the participants were recruited via notices and online communities dealing with different diets. For this reason, a special health awareness of some participants cannot be excluded. Furthermore, the number of participants (*n* = 94) was relatively low as this study was considered to be an exploratory pilot study. The low number of participants could also be the reason for the predominantly weak correlations of relative validity in the present study. However, according to [67], low correlations (rs ≥ 0.2 ≤ 0.3) could already be an indication of significant values for sample sizes with *n* ≤ 250. Furthermore, HEI-flex calculations based on FFQ data, which did not distinguish between rice and whole meal rice or pasta and whole meal pasta, means that the results in this component may be partially inaccurate. Additionally, low processed meat, fish and vegetable protein were combined into one single component: “Protein sources”, regardless of their nutritional-physiological value. The same applies to the foods that are listed in “Fats and oils and plant-based fat substitutes”. Presumably, more differentiated queries, for example, according to brand names or manufacturers, would allow a more accurate assignment of the foods to the HEI-flex components and, thus, more precise results.

### 4.5. Future Research

The present results give first indications of the current dietary quality of FX compared to V and OMN in the German adult population—with FX performing better than OMN but less well than V. Further studies with a larger number of participants and detailed food consumption queries are needed to obtain valid data on dietary quality based on HEI-flex. In addition, the score needs to be validated to ensure resilient reliability in relative and construct validity.

Future prospective studies could also investigate whether associations exist between HEI-flex results and health benefits, for example, through extensive blood analyses. In addition, the inclusion of further sustainability criteria, for example, life cycle assessments, would be a possibility to evaluate diet quality more holistically in the future.

## 5. Conclusions

The HEI-flex enables the first comparison of the nutritional quality between the current common diets of FX, V and OMN in Germany. These results showed that a reduction in meat and meat products seems to be associated with a higher diet quality. However, the partly excessive consumption of processed products, animal- and plant-based, and the high consumption of foods with high-energy density was noticeable in large sections of the study participants. These aspects should be given more attention in the future, especially in the context of possible health consequences [66,68,69,70,71,72,73].

## Figures and Tables

**Table 1:** HEI-flex components, subgroups and evaluation criteria.

Components	Subgroups of the Components	Guideline ^(A)^	Recommendation-Values/ per Day ^(A)^				Calculating Principles: Adequacy or Moderation Principle ^(B)^
			**E_1_**		**E_2_**	**E_3_**	
(1) Beverages	Water, coffee, tea, calorie-reduced beverages, water portion of spritzers	(1), (2)	1500 mL		-.-	-.-	I
(2) Vegetables	Raw and cooked vegetables, max. 1 serving of vegetable juice (≤125 mL)	(1), (2)	400 g		-.-	-.-	I
(3) Fruit	Fresh and cooked fruit, dried fruit, max. 1 portion of fruit juice undiluted (≤125 mL)	(1), (2)	250 g		-.-	-.-	I
(4) Protein sources	Legumes	(1)	125 g		220 g	345 g	II
		(2)	150 g		220 g	370 g	
	Alternative sources of protein unbreaded	(2)	50 g		100 g	150 g	
	Fish, cold and hot, unbreaded	(1)	21 g		31 g	52 g	
	Meat unbreaded	(1)	43 g		64 g	107 g	
	Eggs raw or cooked	(1)	9 g		26 g	35 g	
(5) Carbohydrate sources	Bread/rolls (white, grey and mixed bread)	(1) (2)	200 g		300 g	500 g	II
	Side dishes (noodles, rice, boiled potatoes)		150 g		250 g	400 g	
(6) Whole meal	Muesli, whole meal bread and rolls	(1)	30 g		60 g	90 g	II
(7) Nuts and seeds	Nuts, seeds, sprouts, nut puree	(1)	10 g		25 g	35 g	II
		(2)	30 g		60 g	90 g	
(8) Milk and dairy products and plant-based dairy alternatives	Milk, dairy products, cheese	(1)	250 g		310 g	560 g	II
	Plant-based drinks and plant-based milk alternative products	(2)	200 g		600 g	800 g	
(9) Processed meat and plant-based meat alternatives	Sausage, ham, cold cuts, vegetable-based sausage and meat substitutes and alternative cheese products	(1)	100 g		150 g	350 g	II
(10) Alcohol	Beer, wine, sparkling wine, fruit wine, cocktails, high-proof drinks	(3)	20 cl 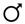	0	-.-	-.-	III
			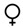 10 cl	0	-.-	-.-	
(11) High-energy density foods (sweet) ^1^	Sweat breakfast cereals, sweet spreads, cakes and biscuits, sweets and chocolate, ice-cream, grain bars, sugar in coffee/tea	(4)	6.5%		26%	-.-	IV
(12) High-energy density foods (fat) ^1^	Salted, fried snacks, convenience or ready-to-eat foods, or plant-based alternatives	(4)	8%		16%	-.-	IV
(13) Drinks with high-energy density	Soft drinks (sugary), 100% fruit/vegetable juices > 125 mL	(3)	192 mL		-.-	-.-	IV
(14) Fats and oils and plant-based fat substitutes	High quality oils (olive, rapeseed, safflower, germ and sunflower oil)	(1)	10 g		15 g	25 g	II
		(2)	24 g		36 g	60 g	
	Solid fats (butter, margarine, vegetable cooking fat, animal fat), spreads and vegetable fats, plant-based alternatives like vegetarian/vegan spreads	(1)	15 g		30 g	45 g	

Overview of the development of the food-based diet quality score HEI-flex with its components, subgroups, official dietary recommendations, limits and evaluation criteria applied, HEI-flex = Healthy Eating Index—flexible.

(A) Official consumption recommendations; (1) Recommendations of the DGE; (2) Recommendations “Gießen Vegan Food Pyramid”; (3) Guideline values World Health Organization; (4) Recommendations dietary guidelines for Americans.

(B) Valuation principles; I Adequacy principle (minimal amount—no limit); II Adequacy principle (minimal amount—consumption range—overconsumption); III Moderation principle (consume as little as possible); IV Moderation principle (tolerated consumption range—overconsumption); E_1_: MIN Recommendation values/per day; E_2_: MAX Recommendation values/per day; E_3_: Up to this value zero SP; ^1^ Recommendations in % of total energy demand.

All components within the HEI-flex were weighted equally; proportional point losses were recorded by under- and overconsumption via the calculating principles.

**Table 2:** Characterization of the study population.

Parameters	Flexitarians (FX)	*p*-Value FX-V *	Vegans (V)	*p*-Value V-OMN **	Omnivores (OMN)	*p*-Value FX-OMN ***	*p*-Value Overall
Total Participants (f = 49/m = 45)	32 (f = 18/m = 14)	-.-	33 (f = 18/m = 15)	-.-	29 (f = 13/m = 16)	-.-	0.633
Age (years)	32 (26–36)	-.-	33 (29–37)	-.-	32 (28–43)	-.-	0.377
Duration of diet		**<0.001**		**<0.001**		**<0.001**	**<0.001**
<5 years, *n*	19		22		1		
6–10 years, *n*	6		9		1		
>11 years, *n*	7		2		27		
Energy expenditure of health-related activities (kcal/week)	3651 (2487–5686)	0.272	5793 (3111–8168)	**0.002**	2681 (1385–4286)	0.250	**0.003**
Body mass index (kg/m^2^)	22 (21–25)	0.375	23 (22–25)	0.223	25 (23–27)	**0.003**	**0.005**
Waist circumference (cm)	74 (71–83)	-.-	78 (72–82)	-.-	78 (76–87)	-.-	0.257
Hip circumference (cm)	99 (93–106)	-.-	101 (95–106)	-.-	103 (98–107)	-.-	0.166
Basal metabolic rate (kcal/day)	1380 (1330–1625)	-.-	1390 (1330–1580)	-.-	1510 (1350–1690)	-.-	0.404

Data are shown as median (25th, 75th percentile), f = female, m = male.

Difference between groups were analyzed using either Kruskal–Wallis with *post hoc* Bonferroni correction (α_adj_ = 0.0167) or Chi-Square test.

*p* > 0.05 was considered significant. *p*-values in bold represent statistical significance

* *p*-value FX-V: not significant or significant values between FX and V.

** *p*-value FX-V: not significant or significant values between V and OMN.

*** *p*-value FX-V: not significant or significant values between FX and OMN.

**Table 3:** Total score results by diet type and gender.

	Flexitarians (FX) *n* = 32 (f/m: 18/14)	*p*-Value FX-V	Vegans (V) *n* = 33 (f/m: 18/15)	*p*-Value V-OMN	Omnivores (OMN) *n* = 29 (f/m: 13/16)	*p*-Value FX-OMN	*p*-Value Overall
**Total Score Points (SP)**							
**Ø**	54 (±8)	**0.010**	61 (±10)	**<0.001**	47 (±9)	**0.008**	**<0.001**
m	52 (49–58)	0.454	60 (50–67)	**0.001**	47 (40–54)	0.139	**0.002**
*p*-value m-f	0.594		0.437		0.442		
f	55 (±9)	0.126	62 (±10)	**0.001**	48 (±11)	0.205	**0.002**
**Components**							
**Beverages**	100 (97–100)	-.-	100 (97–100)	-.-	100 (100)	-.-	0.921
m	100 (100)	-.-	100 (98–100)	-.-	100 (100)	-.-	0.331
*p*-value m-f	0.822		0.479		**0.035**		0.319
f	100 (94–100)	-.-	100 (100)	-.-	100 (83–100)	-.-	0.304
**Vegetables**	71 (36–99)	0.435	94 (48–100)	**<0.001**	27 (17–38)	**0.011**	**<0.001**
m	48 (28–67)	0.480	79 (41–100)	**0.002**	26 (14–38)	0.179	**0.003**
*p*-value m-f	**0.041**		0.506		0.201		**0.012**
f	79 (44–100)	1.000	97 (56–100)	**0.042**	38 (25–62)	0.157	**0.040**
**Fruit**	96 (62–100)	1.000	100 (65–100)	**0.020**	56 (36–100)	0.141	0.136
m	96 (59–100)	-.-	100 (62–100)	-.-	55 (42–92)	-.-	**0.021**
*p*-value m-f	0.951		0.253		0.446		0.147
f	96 (63–100)	-.-	100 (100)	-.-	60 (30–100)	-.-	0.178
**Protein sources**	38 (29–50)	**<0.001**	85 (76–98)	**<0.001**	40 (34–53)	1.000	**<0.001**
m	47 (±17)	**<0.001**	82 (± 25)	**<0.001**	46 (±17)	1.000	**<0.001**
*p*-value m-f	**0.033**		0.222		0.188		0.092
f	32 (22–47)	**<0.001**	80 (68–89)	**0.003**	38 (30–46)	1.000	**<0.001**
**Carbohydrate sources**	46 (22–54)	-.-	50 (20–56)	-.-	44 (37–53)	-.-	0.746
m	50 (36–58)	-.-	55 (29–56)	-.-	44 (42–66)	-.-	0.924
*p*-value m-f	0.133		0.123		0.130		**0.006**
f	35 (21–50)	-.-	38 (17–53)	-.-	41 (21–51)	-.-	0.916
**Whole meal**	41 (0–99)	-.-	62 (0–100)	-.-	71 (15–98)	-.-	0.687
m	4 (0–98)	-.-	12 (0–100)	-.-	80 (50–99)	-.-	0.099
*p*-value m-f	0.122		0.159		0.170		0.361
f	44 (15–100)	-.-	74 (7–100)	-.-	45 (0–92)	-.-	0.617
**Nuts and seeds**	74 (14–100)	-.-	68 (32–100)	-.-	31 (14–100)	-.-	0.426
m	88 (47–100)	-.-	47 (19–100)	-.-	31 (11–62)	-.-	0.087
*p*-value m-f	0.192		0.897		0.564		0.755
f	39 (0–100)	-.-	75 (35–100)	-.-	29 (14–100)	-.-	0.638
**Milk/dairy products and plant-based dairy alternatives**	50 (28–67)	**0.006**	80 (45–100)	0.554	55 (47–89)	0.271	**0.008**
m	44 (± 25)	-.-	65 (± 33)	-.-	58 (± 17)	-.-	0.204
*p*-value m-f	0.569		0.362		0.693		0.336
f	52 (28–76)	**0.035**	100 (51–100)	0.627	55 (50–89)	0.876	**0.041**
**Processed meat and plant-based meat alternatives**	17 (5–60)	-.-	38 (13–77)	-.-	5 (0–74)	-.-	0.122
m	28 (13–92)	-.-	52 (15–92)	-.-	0 (0–67)	-.-	0.091
*p*-value m-f	0.136		0.232		0.545		0.289
f	13 (0–36)	-.-	37 (10–77)	-.-	20 (0–74)	-.-	0.549
**Alcohol**	70 (19–96)	0.463	91 (41–100)	**0.013**	46 (17–77)	0.446	**0.018**
m	87 (49–98)	1.000	69 (41–100)	0.052	41 (21–73)	0.072	**0.026**
*p*-value m-f	0.082		0.795		0.660		0.632
f	43 (4–78)	-.-	70 (38–100)	-.-	50 (2–84)	-.-	0.152
**High-energy density foods (sweet)**	0 (0–33)	0.527	11 (0–52)	**0.037**	0 (0–10)	0.722	**0.043**
m	0 (0–27)	0.114	28 (0–56)	**0.029**	0 (0–5)	1.000	**0.023**
*p*-value m-f	0.305		0.198		0.738		0.875
f	2 (0–46)	-.-	1 (0–40)	-.-	0 (0–18)	-.-	0.523
**High-energy density foods (fat)**	38 (0–100)	1.000	35 (0–85)	**0.010**	0 (0)	**0.012**	**0.004**
m	0 (0–47)	0.713	12 (0–70)	**0.036**	0 (0)	0.614	**0.043**
*p*-value m-f	**0.035**		0.600		0.307		**0.021**
f	65 (0–100)	1.000	47 (0–85)	0.302	0 (0–34)	**0.040**	**0.045**
**Drinks with high-energy density**	96 (78–100)	-.-	91 (78–100)	-.-	78 (0–96)	-.-	0.092
m	80 (39–96)	-.-	91 (76–100)	-.-	52 (0–92)	-.-	0.087
*p*-value m-f	**0.016**		0.713		0.142		**0.007**
f	96 (91–100)	-.-	94 (78–100)	-.-	96 (39–100)	-.-	0.471
**Fats and oils and plant-based fat substitutes**	62 (± 20)	-.-	48 (±27)	-.-	59 (± 24)	-.-	0.087
m	57 (42–76)	-.-	57 (25–63)	-.-	61 (54–76)	-.-	0.266
*p*-value m-f	0.621		0.690		0.677		0.982
f	55 (50–81)	-.-	48 (25–75)	-.-	53 (40–79)	-.-	0.184

FX = flexitarians, V = vegans, OMN = omnivores. f = female, m = male. *n* = number of participants. SP = score points; V_max_ = 100 SP, V_min_ = 0 SP. Normally distributed data are shown as mean Ø ± SD and not normally distributed data as median $\tilde{x}$ with 25th, 75th percentile. The difference between groups were analyzed using either univariate ANOVA for normally distributed data or the Kruskal–Wallis test for not-normally distributed data; *post hoc* Bonferroni correction (α_adj_ = 0.0167). Difference between sex were analyzed using the Mann–Whitney U test. *p* > 0.05 was considered significant. *p*-values in bold represent statistical significance.

**Table 4:** Indications of relative validity: Nutrient intake of three-day food record in correlation to selected HEI-flex components.

	Total Score	*p*_rs_-Value	Score: Carbo-hydrate Sources	*p*_rs_-Value	Score: Protein Sources	*p*_rs_-Value	Score: Whole Meal	*p*_rs_-Value	Score: Beverages	*p*_rs_-Value	Score: Alcohol	*p*_rs_-Value	Score: High-energy Density Foods (Sweet)	*p*_rs_-Value	Score: High-Energy Density Foods (Fat)	*p*_rs_-Value	Score: Drinks with High-Energy Density	*p*_rs_-Value
Total Energy [kJ]	0.206	**0.048**	0.017	0.872	−0.063	0.550	−0.260	**0.012**	0.072	0.493	0.060	0.566	−0.027	0.800	−0.013	0.902	−0.125	0.234
Protein [g]	0.158	0.129	−0.042	0.690	0.024	0.822	−0.060	0.565	0.131	0.211	−0.043	0.680	0.046	0.663	0.021	0.844	−0.062	0.555
Carbohydrates [g]	0.146	0.162	−0.098	0.348	0.087	0.405	−0.254	**0.014**	−0.032	0.757	0.217	**0.036**	0.041	0.698	0.084	0.423	−0.128	0.221
Total fat [g]	0.229	**0.027**	0.129	0.217	−0.064	0.542	−0.292	**0.005**	0.025	0.810	−0.072	0.494	−0.090	0.389	−0.084	0.442	−0.056	0.595
Dietary fiber [g]	0.477	**<0.001**	−0.256	**0.013**	0.429	**<0.001**	−0.187	0.073	0.037	0.722	0.312	**0.002**	0.437	**<0.001**	0.369	**<0.001**	0.314	**0.002**
Total minerals [g]	0.045	0.668	−0.057	0.585	0.065	0.536	−0.175	0.093	0.217	**0.037**	−0.008	0.942	0.083	0.429	0.077	0.464	0.033	0.753
Sodium [g]	0.366	**<0.001**	0.311	**0.002**	−0.161	0.122	−0.38	0.717	−0.029	0.786	−0.283	**0.006**	−0.212	0.041	−0.254	**0.014**	−0.216	**0.039**
Water [g]	0.220	**0.034**	−0.031	0.768	0.098	0.351	0.150	0.151	0.325	**0.001**	−0.069	0.509	0.342	**0.001**	0.140	0.180	0.119	0.257
Alcohol [g]	0.309	**0.003**	0.215	**0.038**	−0.071	0.496	0.146	0.163	0.261	**0.012**	−0.495	**<0.001**	−0.208	**0.045**	−0.215	**0.038**	−0.114	0.275

Spearman’s rank correlation coefficient (rs) is standardized on the interval (−1, +1) with two-sided *p*_rs_-values.

*p*_rs_ < 0.05 = significant. *p*_rs_ < 0.01 = high significant.

HEI-flex: Healthy Eating Index—flexible *p*-values in bold represent statistical significance.

**Table 5:** Indications of construct validity: Macronutrient intake and cardiovascular risk parameters according to distribution thirds of HEI-flex.

Energy and Nutrients/Day	Thirds of HEI-Flex			*p*-Value
	Low (0–49.0)	Middle (49.0–60.0)	Upper (≥60.0)	
Energy [kJ]	9438.53 (8197.46–10.503.02) ^b^	9500.03 (8392.06–11,037.16) ^a^	8371.81 (6884.13–9045.85) ^a,b^	**0.043**
Protein [%]	15.14 (12.81–16.12)	14.68 (12.99–17.79)	14.19 (12.85–15.59)	0.069
Carbohydrates [%]	43.63 (±7.88)	40.67 (±8.14)	44.98 (±10.06)	0.153
Total fat [%]	36.63 (±7.78)	38.67 (±8.04)	35.76 (±9.78)	0.405
Dietary fiber [g]	22.56 (14.46–28.47) ^b^	28.30 (19.27–35.90) ^a^	34.60 (26.74–47.79) ^a,b^	**<0.001**
Salt [g]	5.22 (±2.16) ^b^	5.38 (±2.25) ^a^	3.36 (±1.83) ^a,b^	**<0.001**
Water [g]	2596.26 (±968.24) ^b^	2962.29 (±888.46)	3065.38 (±1164.22) ^b^	**0.048**
Alcohol [g]	4.72 (0–13.16) ^b^	2.54 (0–8.91) ^a^	0.03 (0–0.95) ^a,b^	**0.006**
Body fat [%]	27.99 (±7.85)	27.40 (±7.04)	27.44 (±6.60)	0.936
Trigyceride [mmol/l]	0.90 (0.79–1.32)	0.81 (0.62–1.28)	0.71 (0.59–0.98)	0.056
Insulin [mU/l]	7.00 (5.40–10.50) ^b^	5.50 (3.70–6.80) ^a^	5.40 (4.20–6.80) ^a,b^	**0.013**
HDL-Cholesterin [mmol/l]	1.50 (±0.38)	1.62 (±0.31)	1.70 (±0.36)	0.087
LDL-Cholesterin [mmol/l]	2.65 (2.46–3.53)	2.57 (1.97–3.24)	2.33 (2.01–2.77)	0.054
Systolic blood pressure [mmHg]	125 (121–132)	126 (120–137)	123 (119–130)	0.342
Diastolic blood pressure [mmHg]	77 (±8)	77 (±8)	75 (±7)	0.514

Data are shown as mean ± SD (normally distributed) or median with 25th, 75th percentile (not normally distributed). Differences between the thirds of the total study population regarding macronutrient intake and cardiovascular risk parameters were analyzed using ANOVA or the Kruskal–Wallis test with *post hoc* Bonferroni correction (α_adj_ = 0.0167).

*p* ≤ 0.05 = significant; n.s. = not significant. *p*-values in bold represent statistical significance.

^a^ = significant difference between the thirds “middle” and “upper”. ^b^ = significant difference between the thirds “low” and “upper”. HEI-flex = Healthy Eating Index—flexible.

## Data Availability

Additional information about the study are available upon request from the corresponding author.

## Appendix A

Supplements to the food frequency questionnaire

Main focus: Plant-based alternatives

(58) How often have you drunk **plant-based milk drinks** (including plant-based milk for coffee, muesli) **in the last 4 weeks**?

O Never (please continue with question 59)	
O 1 time per month	O 1 per day
O 2–3 times per month	O 2 per day
O 1–2 per week	O 3 per day
O 3–4 per week	O 4–5 per day
O 5–6 per week	O more than 5 times per day

(58a) If you drink **plant-based milk drinks**, **how much** do you drink of them most of the time?

O ½ glass (or less)
O 1 glass (200 mL)
O 2 glasses
O 3 glasses
O 4 glasses (or more)

(58b) What kind of **plant-based milk drinks** do you drink most often?

O Oat drink
O Soy drink
O Almond drink
O Rice drink
O Coconut drink

(59) How often have you eaten **vegan cream cheese in the last 4 weeks**?

O Never (please continue with question (60)	
O 1 time per month	O 1 per day
O 2–3 times per month	O 2 per day
O 1–2 per week	O 3 per day
O 3–4 per week	O 4–5 per day
O 5–6 per week	O more than 5 times per day

(59a) When you eat **vegan cream cheese**, **how much** do you eat of it most of the time?

O ½ tablespoon (or less)
O 1 tablespoon (spread)
O 2 tablespoon (spread)
O 3 tablespoon (spread)
O 4 tablespoon (spread)

(60) How often have you eaten **vegan cheese** (soft, semi-hard or hard) **in the last 4 weeks**?

O Never (please continue with question (61)	
O 1 time per month	O 1 per day
O 2–3 times per month	O 2 per day
O 1–2 per week	O 3 per day
O 3–4 per week	O 4–5 per day
O 5–6 per week	O more than 5 times per day

(60a) When you eat **vegan cheese**, **how much** do you eat of it most of the time?

O ½ slice or ½ portion (or less)
O 1 slice or 1 portion
O 2 slices or 2 portions
O 3 slices or 3 portions
O 4 slices or 4 portions (or more)

(61) How often have you eaten **vegan yoghurt** (based on soy, cashew, coconut, almond, macadamia, etc.) **in the last 4 weeks**?

O Never (please continue with question 62)	
O 1 time per month	O 1 per day
O 2–3 times per month	O 2 per day
O 1–2 per week	O 3 per day
O 3–4 per week	O 4–5 per day
O 5–6 per week	O more than 5 times per day

(61a) When you eat **vegan yoghurt**, **how much** do you eat of it most of the time?

O ½ cup (or less)
O 1 cup (200 g)
O 2 cups
O 3 cups
O 4 cups

(62) How often have you eaten vegan/vegetarian **meat alternatives** (tofu, ready-made products, etc.) **in the last 4 weeks** (e.g., based on soy, cereals, mushrooms, pulses, tempeh, seitan)?

O Never (please continue with question (63)	
O 1 time per month	O 1 per day
O 2–3 times per month	O 2 per day
O 1–2 per week	O 3 per day
O 3–4 per week	O 4–5 per day
O 5–6 per week	O more than 5 times per day

(62a) If you eat vegan/vegetarian **meat alternatives**, **how much** do you eat of them most of the time?

O ¼ portion (or less)
O ½ portion
O 1 portion
O 2 portions
O 3 servings (or more)

(62b) **How often** were the **vegan/vegetarian meat alternatives** ready-made products (e.g., vegetable schnitzels, sausages, gyros)?

O (Almost) never
O About ¼ of the consumption
O About ½ of the consumption
O About ¾ of the consumption
O (Almost) always

(63) How often have you eaten **vegan/vegetarian sausage** (mortadella, salami, etc.) **in the last 4 weeks**?

O Never (please continue with question (64)	
O 1 time per month	O 1 per day
O 2–3 time per month	O 2 per day
O 1–2 per week	O 3 per day
O 3–4 per week	O 4–5 per day
O 5–6 per week	O more than 5 times per day

(63a) If you eat **vegan/vegetarian sausage**, **how much** do you eat of it most of the time?

O ½ disc
O 1 disc
O 2 discs
O 3 discs
O 4 discs (or more)

(64) How often have you eaten **vegan/vegetarian spreads** made from vegetables or pulses (including hummus) **in the last 4 weeks**?

O Never (please continue with question (65)	
O 1 time per month	O 1 per day
O 2–3 times per month	O 2 per day
O 1–2 per week	O 3 per day
O 3–4 per week	O 4–5 per day
O 5–6 per week	O more than 5 times per day

(64a) If you eat **vegan/vegetarian spread**, **how much** do you eat of it most of the time?

O 1 teaspoon (or less)
O 2 teaspoons (heaped)
O 3 teaspoons (heaped)
O 4 teaspoons (heaped)
O 5 teaspoons (heaped)

(65) How often have you eaten **nut puree/cream in the last 4 weeks**?

O Never (please continue with question (66)	
O 1 time per month	O 1 per day
O 2–3 times per month	O 2 per day
O 1–2 per week	O 3 per day
O 3–4 per week	O 4–5 per day
O 5–6 per week	O more than 5 times per day

(65a) When you eat **nut puree/cream**, **how much** do you eat of it most of the time?

O 1 teaspoon (or less)
O 2 teaspoons (heaped)
O 3 teaspoons (heaped)
O 4 teaspoons (heaped)
O 5 teaspoons (heaped)

(66) How often have you eaten **dried fruits** (e.g., sultanas) **in the last 4 weeks**?

O Never (please continue with question (67)	
O 1 time per month	O 1 per day
O 2–3 times per month	O 2 per day
O 1–2 per week	O 3 per day
O 3–4 per week	O 4–5 per day
O 5–6 per week	O more than 5 times per day

(66a) When you eat **dried fruit**, **how much** do you eat most of the time?

O ½ tablespoon (or less)
O 1 tablespoon
O 2 tablespoons
O 3 tablespoons
O 4 tablespoons

(67) How often have you eaten **seeds** (e.g., linseed, pumpkin seeds, sesame seeds) **in the last 4 weeks**?

O Never (please continue with question 68)	
O 1 time per month	O 1 per day
O 2–3 times per month	O 2 per day
O 1–2 per week	O 3 per day
O 3–4 per week	O 4–5 per day
O 5–6 per week	O more than 5 times per day

(67a) When you eat **seeds**, **how much** do you eat of them most of the time?

O ½ tablespoon (or less)
O 1 tablespoon
O 2 tablespoons
O 3 tablespoons
O 4 tablespoons

(68) How often have you eaten **sprouts in the last 4 weeks**?

O Never (please continue with question (69)	
O 1 time per month	O 1 per day
O 2–3 times per month	O 2 per day
O 1–2 per week	O 3 per day
O 3–4 per week	O 4–5 per day
O 5–6 per week	O more than 5 times per day

(68a) If you eat **sprouts/shoots**, **how much** do you eat of them most of the time?

O ¼ portion (or less)
O ½ portion
O 1 portion
O 2 portions
O 3 servings (or more)

(69) How often have you eaten **energy bars in the last 4 weeks**?

O Never (please continue with question (70)	
O 1 time per month	O 1 per day
O 2–3 times per month	O 2 per day
O 1–2 per week	O 3 per day
O 3–4 per week	O 4–5 per day
O 5–6 per week	O more than 5 times per day

(69a) When you eat **energy bars**, **how much** do you eat of them most of the time?

O ¼ bar (or less)
O ½ bar
O 1 bar
O 2 bars
O 3 bars (or more)

(70) How often did you drink **liquid food/complete food in the last 4 weeks**?

O Never (please finish this questionnaire)	
O 1 time per month	O 1 per day
O 2–3 time per month	O 2 per day
O 1–2 per week	O 3 per day
O 3–4 per week	O 4–5 per day
O 5–6 per week	O more than 5 times per day

(70a) If you drink **liquid food/complete food**, **how much** of it do you drink most of the time?

O ½ cup (or less)
O 1 cup (200 mL)
O 2 cups
O 3 cups
O 4 cups (or more)

**Thank you very much!**

## Appendix B

**Table A1:** Valuation principles.

**Adequacy Principles**		
Adequacy Principle Type I:	V/E ≤ 1 = Points ↑ V/E > 1 = 100 Points	
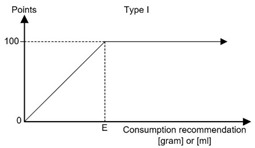
Adequacy Principle Type II:	V/E ≤ 1 = Points ↑ V/E > 1 ≤ 2 = 100 Points V/E > 2 = Points ↓	
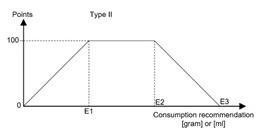
**Moderation Principles**		
Moderation Principle Type III:	V/E = 0 = 100 Points V/E > 0 ≤ 1 = Points ↓	
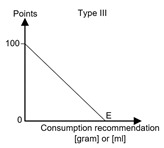
Moderation Principle Type IV:	V/E = 0 = 100 Points V/E > 0 ≤ 1 = 100 Points V/E > 1 = Points ↓	
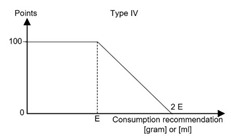

Assessment principles of the HEI-flex components; HEI-flex = Healthy Eating Index—flexible.

↑ points increase; ↓ points decrease.

## Appendix C

**Table A2:** Essential similarities and differences of HEI-flex and HEI-2015.

Aspects	HEI-Flex	HEI-2015
Able to differentiate between dietary pattern Deposited Guidelines	yes (flexitarians, vegans, omnivores) DGE, Vegan Pyramid (for components 1–9, 14) WHO (for components 10 and 13) DGA (for 11 and 12)	no (omnivores) DGA for all components:
Units	gram, milliliter, % of total energy	cup, ounce, % of total energy
Considers as separate component:		
-beverages (non-caloric)	yes	no
-alcohol	yes	no
-drinks with high-energy density	yes	no
Considers processing of the foods	yes	no
Considers variety of plant-based alternatives	yes	no
Scoring system:		
-weighting of components equally	yes	no
-minimum and maximum values	V_min_ = 0; V_max_ = 100 SP (for each component)	V_min_ = 0; V_max_ between 5 and 10 SP (depending on component)
-considers overconsumption	yes	partly
-considers individual total energy expenditure	yes	no (uses standard values)

Own compilation of HEI-flex aspects and according to [52]. DGE= Deutsche Gesellschaft für Ernährung (German Nutrition Society). DGA = Dietary guidelines for Americans. WHO = World Health Organization. V_min_ = maximum value. V_max_ = minimum value. SP = Score Points.

## References

[1] Gallup Poll Research Few Americans Vegetarian or Vegan. https://news.gallup.com/poll/238328/snapshot-few-americans-vegetarian-vegan.aspx.

[2] Roy Morgan Resaearch The Slow but Steady Rise of Vegetarianism in Australia. http://www.roymorgan.com/findings/vegetarianisms-slow-but-steady-rise-in-australia-201608151105.

[3] Ipsos Diets around the World: An Exploration. https://www.ipsos.com/en/diets-around-world-exploration.

[4] Derbyshire E.J. (2017). Flexitarian Diets and Health: A Review of the Evidence-Based Literature. Front. Nutr..

[5] GfK (2016). GFK-Studie zum Fleischkonsum: Ein Drittel der Haushalte Hält Sich für Flexitarier. https://www.gfkverein.org/sites/default/files/medien/1288/dokumente/ci_03_2016_od.pdf.

[6] Veganz (2022). Veganz Ernährungsstudie 2021–Das essen die Europäer!. https://veganz.com/blog/results-of-the-current-veganz-nutrition-study.

[7] Dietary Choices of Brits (e.g. Vegeterian, Flexitarian, Meat-Eater etc)?. https://yougov.co.uk/topics/lifestyle/trackers/dietery-choices-of-brits-eg-vegeterian-flexitarian-meat-eater-etc.

[8] Dawczynski C. (2021). A Study Protocol for a Parallel-Designed Trial Evaluating the Impact of Plant-Based Diets in Comparison to Animal-Based Diets on Health Status and Prevention of Non-communicable Diseases—The Nutritional Evaluation (NuEva) Study. Front. Nutr..

[9] Papier K., Tong T.Y., Appleby P.N., Bradbury K.E., Fensom G.K., Knuppel A., Perez-Cornago A., Schmidt J.A., Travis R.C., Key T.J. (2019). Comparison of Major Protein-Source Foods and Other Food Groups in Meat-Eaters and Non-Meat-Eaters in the EPIC-Oxford Cohort. Nutrients.

[10] Springmann M., Wiebe K., Mason-D’Croz D., Sulser T.B., Rayner M., Scarborough P. (2018). Health and nutritional aspects of sustainable diet strategies and their association with environmental impacts: A global modelling analysis with country-level detail. Lancet Planet. Health.

[11] Flexitarier—Die Flexiblen Vegetarier. https://www.dge.de/wissenschaft/weitere-publikationen/fachinformationen/flexitarier-die-flexiblen-vegetarier/?.

[12] 10 Regeln der DGE für Eine Vollwertige Ernährung Überarbeitet. https://www.dge.de/presse/pm/10-regeln-der-dge-fuer-eine-vollwertige-ernaehrung-ueberarbeitet.

[13] Choudhury D., Singh S., Seah J.S.H., Yeo D.C.L., Tan L.P. (2020). Commercialization of Plant-Based Meat Alternatives. Trends Plant Sci..

[14] Pointke M., Pawelzik E. (2022). Plant-Based Alternative Products: Are They Healthy Alternatives? Micro- and Macronutrients and Nutritional Scoring. Nutrients.

[15] Heinrich-Böll-Stiftung Fleischalternativen: Vegetarischer und Veganer Fleischersatz Wächst. https://www.boell.de/de/2021/01/06/fleischalternativen-vegetarischer-und-veganer-fleischersatz-waechst.

[16] Smart Protein Project Plant-Based Foods in Europe: How Big Is the Market?. https://smartproteinproject.eu/plant-based-food-sector-report/.

[17] BMEL (2019). Der BMEL-Ernährungsreport. https://www.in-form.de/wissen/der-bmel-ernaehrungsreport-2019/.

[18] BMEL Deutschland, wie es isst-der BMEL-Ernährungsreport 2020. https://www.bmel.de/DE/themen/ernaehrung/ernaehrungsreport2020.html.

[19] Hu F.B., Otis B.O., McCarthy G. (2019). Can Plant-Based Meat Alternatives Be Part of a Healthy and Sustainable Diet?. JAMA.

[20] Koch F., Heuer T., Krems C., Claupein E. (2019). Meat consumers and non-meat consumers in Germany: A characterisation based on results of the German National Nutrition Survey II. J. Nutr. Sci..

[21] Onwezen M.C., Bouwman E.P., Reinders M.J., Dagevos H. (2021). A systematic review on consumer acceptance of alternative proteins: Pulses, algae, insects, plant-based meat alternatives, and cultured meat. Appetite.

[22] BMEL BMEL Deutschland, wie es isst-der BMEL-Ernährungsreport 2021. https://www.bmel.de/DE/themen/ernaehrung/ernaehrungsreport2021.html.

[23] Gebhardt B., Hadwiger K. (2020). Plant-based foods for future. Results of consumer and professional expert interviews in five European countries-EIT-Food Project “The V-Place”. Hohenheimer Agrarökonomische Arbeitsberichte.

[24] Vegetarismus und Flexitarismus. https://www.splendid-research.com/de/studie-vegetarier-flexitarier.

[25] Kennedy E.T., Ohls J., Carlson S., Fleming K., Kennedy E.T., Ohls J., Carlson S., Fleming K., Kennedy E.T., Ohls J. (1995). The Healthy Eating Index: Design and Applications. J. Am. Diet. Assoc..

[26] Dietary Guidelines for Americans. https://www.dietaryguidelines.gov.

[27] Jailani M., Elias S.M., Rajikan R. (2021). The New Standardized Malaysian Healthy Eating Index. Nutrients.

[28] Kyttälä P., Erkkola M., Lehtinen-Jacks S., Ovaskainen M.-L., Uusitalo L., Veijola R., Simell O., Knip M., Virtanen S.M. (2013). Finnish Children Healthy Eating Index (FCHEI) and its associations with family and child characteristics in pre-school children. Public Health Nutr..

[29] Looman M., Feskens E.J., De Rijk M., Meijboom S., Biesbroek S., Temme E.H., De Vries J., Geelen A. (2017). Development and evaluation of the Dutch Healthy Diet index 2015. Public Health Nutr..

[30] Roy R., Hebden L., Rangan A., Allman-Farinelli M. (2016). The development, application, and validation of a Healthy eating index for Australian Adults (HEIFA—2013). Nutrition.

[31] Taechangam S., Pinitchun U., Pachotikarn C. (2008). Development of nutrition education tool: Healthy eating index in Thailand. Asia Pac. J. Clin. Nutr..

[32] Woodruff S.J., Hanning R.M. (2010). Development and implications of a revised Canadian Healthy Eating Index (HEIC-2009). Public Health Nutr..

[33] Yuan Y.-Q., Li F., Dong R.-H., Chen J.-S., He G.-S., Li S.-G., Chen B. (2017). The Development of a Chinese Healthy Eating Index and Its Application in the General Population. Nutrients.

[34] von Ruesten A., Illner A.-K., Buijsse B., Heidemann C., Boeing H. (2010). Adherence to recommendations of the German food pyramid and risk of chronic diseases: Results from the EPIC-Potsdam study. Eur. J. Clin. Nutr..

[35] (2009). Die Bewertung der Lebensmittelaufnahme mittels eines,Healthy Eating Index‘ (HEI-EPIC) (Peer-Review-Beitrag). https://www.ernaehrungs-umschau.de/print-artikel/13-08-2009-die-bewertung-der-lebensmittelaufnahme-mittels-eines-healthy-eating-index-hei-epic-peer-review-beitrag.

[36] Hoffmann I. Auswertung der Daten der Nationalen Verzehrsstudie II (NVS II): Eine integrierte verhaltens- und lebensstilbasierte Analyse des Bio-Konsums. Data Interpretation Based on the German National Nutrition Survey II (NVS II): An Integrative Analysis of Behavioural and Lifestyle-Related Factors for Organic Food Consumption. http://forschung.oekolandbau.de.

[37] Blaurock J., Kaiser B., Stelzl T., Weech M., Fallaize R., Franco R., Hwang F., Lovegrove J., Finglas P., Gedrich K. (2021). Dietary Quality in Vegetarian and Omnivorous Female Students in Germany: A Retrospective Study. Int. J. Environ. Res. Public Health.

[38] Kuhn D.-A. (2018). Entwicklung Eines Index zur Bewertung der Ernährungsqualität in der Studie zur Gesundheit Erwachsener in Deutschland (DEGS1). Master’s Thesis.

[39] Heinrich-Böll-Stiftung Fleischatlas 2018. https://www.boell.de/de/2018/01/10/fleischatlas-2018-rezepte-fuer-eine-bessere-tierhaltung.

[40] Spiller A., Cordts A., Nitzko S., Grethe H., Duman N. (2016). Imageprobleme Beeinflussen Fleischkonsum. https://www.fleischwirtschaft.de/wirtschaft/nachrichten/Imageprobleme-beeinflussen-Fleischkonsum--20426.

[41] Eckart W.U., Gradmann C. (2006). Ärzte Lexikon.

[42] Eknoyan G. (2007). Adolphe Quetelet (1796 1874) the average man and indices of obesity. Nephrol. Dial. Transplant..

[43] Frey I., Berg A., Grathwohl D., Keul J. (1999). Freiburger Fragebogen zur körperlichen Aktivität-Entwicklung, Prüfung und Anwendung. Soz.-Und Präventivmed..

[44] Haftenberger M., Heuer T., Heidemann C., Kube F., Krems C., Mensink G.B. (2010). Relative validation of a food frequency questionnaire for national health and nutrition monitoring. Nutr. J..

[45] Fokeena W.B., Jamaluddin R., Khaza’ai H. (2016). Development and Assessment of the Reliability and Validity of a Diet Quality Index in a Sample of Malaysian University Students. J. Food Nutr. Res..

[46] Panizza C.E., Shvetsov Y.B., Harmon B.E., Wilkens L.R., Le Marchand L., Haiman C., Reedy J., Boushey C.J. (2018). Testing the Predictive Validity of the Healthy Eating Index-2015 in the Multiethnic Cohort: Is the Score Associated with a Reduced Risk of All-Cause and Cause-Specific Mortality?. Nutrients.

[47] Reedy J., Lerman J.L., Krebs-Smith S.M., Kirkpatrick S.I., Pannucci T.E., Wilson M.M., Subar A.F., Kahle L.L., Tooze J.A. (2018). Evaluation of the Healthy Eating Index-2015. J. Acad. Nutr. Diet..

[48] Wolfson J.A., Leung C.W., Richardson C.R. (2020). More frequent cooking at home is associated with higher Healthy Eating Index-2015 score. Public Health Nutr..

[49] Truthmann J., Mensink G.B., Richter A. (2011). Relative validation of the KiGGS Food Frequency Questionnaire among adolescents in Germany. Nutr. J..

[50] Weder S., Keller M., Schaefer C. (2018). Die Gießener Vegane Lebensmittelpyramide. https://www.ernaehrungs-umschau.de/print-artikel/15-08-2018-die-giessener-vegane-lebensmittelpyramide.

[51] Kanauchi M., Kanauchi K. (2018). The World Health Organization’s Healthy Diet Indicator and its associated factors: A cross-sectional study in central Kinki, Japan. Prev. Med. Rep..

[52] Krebs-Smith S.M., Pannucci T.E., Subar A.F., Kirkpatrick S.I., Lerman J.L., Tooze J.A., Wilson M.M., Reedy J. (2018). Update of the Healthy Eating Index: HEI-2015. J. Acad. Nutr. Diet..

[53] Clarys P., Deliens T., Huybrechts I., Deriemaeker P., Vanaelst B., De Keyzer W., Hebbelinck M., Mullie P. (2014). Comparison of Nutritional Quality of the Vegan, Vegetarian, Semi-Vegetarian, Pesco-Vegetarian and Omnivorous Diet. Nutrients.

[54] Conrad Z., Karlsen M., Chui K., Jahns L. (2017). Diet quality on meatless days: National Health and Nutrition Examination Survey (NHANES), 2007–2012. Public Health Nutr..

[55] Farmer B., Larson B.T., Fulgoni V.L., Rainville A.J., Liepa G.U. (2011). A vegetarian dietary pattern as a nutrient-dense approach to weight management: An analysis of the national health and nutrition examination survey 1999–2004. J. Am. Diet. Assoc..

[56] Kennedy E.T., Bowman S.A., Spence J.T., Freedman M., King J. (2001). Popular Diets. J. Am. Diet. Assoc..

[57] Rosi A., Mena P., Pellegrini N., Turroni S., Neviani E., Ferrocino I., Di Cagno R., Ruini L., Ciati R., Angelino D. (2017). Environmental impact of omnivorous, ovo-lacto-vegetarian, and vegan diet. Sci. Rep..

[58] Toft U., Kristoffersen L.H., Lau C., Borch-Johnsen K., Jørgensen T. (2006). The Dietary Quality Score: Validation and association with cardiovascular risk factors: The Inter99 study. Eur. J. Clin. Nutr..

[59] Turner-McGrievy G.M., Barnard N.D., Cohen J., Jenkins D.J., Gloede L., Green A.A. (2008). Changes in Nutrient Intake and Dietary Quality among Participants with Type 2 Diabetes Following a Low-Fat Vegan Diet or a Conventional Diabetes Diet for 22 Weeks. J. Am. Diet. Assoc..

[60] Van Duong T., Tseng I.-H., Wong T.-C., Chen H.-H., Chen T.-H., Hsu Y.-H., Peng S.-J., Kuo K.-L., Liu H.-C., Lin E.-T. (2019). Adaptation and Validation of Alternative Healthy Eating Index in Hemodialysis Patients (AHEI-HD) and Its Association with all-Cause Mortality: A Multi-Center Follow-Up Study. Nutrients.

[61] Murakami K., Livingstone M.B.E., Fujiwara A., Sasaki S. (2020). Application of the Healthy Eating Index-2015 and the Nutrient-Rich Food Index 9.3 for assessing overall diet quality in the Japanese context: Different nutritional concerns from the US. PLoS ONE.

[62] Parker H.W., Vadiveloo M.K. (2019). Diet quality of vegetarian diets compared with nonvegetarian diets: A systematic review. Nutr. Rev..

[63] Souza J.D.P.M., de Lima M.M., Horta P.M. (2019). Diet Quality among the Brazilian Population and Associated Socioeconomic and Demographic Factors: Analysis from the National Dietary Survey 2008–2009. J. Acad. Nutr. Diet..

[64] Wong J.E., Parnell W.R., Howe A.S., Black K.E., Skidmore P.M. (2013). Development and validation of a food-based diet quality index for New Zealand adolescents. BMC Public Health.

[65] The Nutrition Source (2015). WHO Report Says Eating Processed Meat Is Carcinogenic: Understanding the Findings. https://www.hsph.harvard.edu/nutritionsource/2015/11/03/report-says-eating-processed-meat-is-carcinogenic-understanding-the-findings.

[66] (2015). IARC Monographs Evaluate Consumption of Red Meat and Processed Meat. http://www.iarc.fr/en/media-centre/iarcnews/pdf/Monographs-Q&A_Vol114.pdf.

[67] Schönbrodt F., Perugini M. (2013). At what sample size do correlations stabilize?. J. Res. Personal..

[68] Richi E.B., Baumer B., Conrad B., Darioli R., Schmid A., Keller U. (2015). Health Risks Associated with Meat Consumption: A Review of Epidemiological Studies. Int. J. Vitam. Nutr. Res..

[69] Neuschwander-Tetri B.A. (2019). Too Much Sugar—The Not-So-Sweet Reality of Its Impact on Our Health. Hepatology.

[70] Reedy J., Mitrou P.N., Krebs-Smith S.M., Wirfält E., Flood A., Kipnis V., Leitzmann M., Mouw T., Hollenbeck A., Schatzkin A. (2008). Index-based Dietary Patterns and Risk of Colorectal Cancer: The NIH-AARP Diet and Health Study. Am. J. Epidemiol..

[71] SSofi F., Cesari F., Abbate R., Gensini G.F., Casini A. (2008). Adherence to Mediterranean diet and health status: Meta-analysis. BMJ.

[72] McCullough M.L., Feskanich D., Stampfer M.J., Giovannucci E.L., Rimm E.B., Hu F.B., Spiegelman D., Hunter D.J., Colditz G., Willett W.C. (2002). Diet quality and major chronic disease risk in men and women: Moving toward improved dietary guidance. Am. J. Clin. Nutr..

[73] Han M.A., Zeraatkar D., Guyatt G.H., Vernooij R.W., El Dib R., Zhang Y., Algarni A., Leung G., Storman D., Valli C. (2019). Reduction of Red and Processed Meat Intake and Cancer Mortality and Incidence. Ann. Intern. Med..

